# Asymmetric warming significantly affects net primary production, but not ecosystem carbon balances of forest and grassland ecosystems in northern China

**DOI:** 10.1038/srep09115

**Published:** 2015-03-13

**Authors:** Hongxin Su, Jinchao Feng, Jan C. Axmacher, Weiguo Sang

**Affiliations:** 1 State Key Laboratory of Vegetation and Environmental Change, Institute of Botany, Chinese Academy of Sciences, Beijing 100093, P.R. China; 2 College of Life and Environmental Science, Minzu University of China, 100081 Beijing, P.R. China; 3 UCL Department of Geography, University College London, Pearson Building, Gower Street, London WC1E 6BT, UK

## Abstract

We combine the process-based ecosystem model (Biome-BGC) with climate change-scenarios based on both RegCM3 model outputs and historic observed trends to quantify differential effects of symmetric and asymmetric warming on ecosystem net primary productivity (NPP), heterotrophic respiration (R_h_) and net ecosystem productivity (NEP) of six ecosystem types representing different climatic zones of northern China. Analysis of covariance shows that NPP is significant greater at most ecosystems under the various environmental change scenarios once temperature asymmetries are taken into consideration. However, these differences do not lead to significant differences in NEP, which indicates that asymmetry in climate change does not result in significant alterations of the overall carbon balance in the dominating forest or grassland ecosystems. Overall, NPP, R_h_ and NEP are regulated by highly interrelated effects of increases in temperature and atmospheric CO_2_ concentrations and precipitation changes, while the magnitude of these effects strongly varies across the six sites. Further studies underpinned by suitable experiments are nonetheless required to further improve the performance of ecosystem models and confirm the validity of these model predictions. This is crucial for a sound understanding of the mechanisms controlling the variability in asymmetric warming effects on ecosystem structure and functioning.

Historical observations over a large section of the earth's land area suggest that minimum temperatures (T_min_) have increased significantly faster than maximum temperatures (T_max_) since 1950 - a phenomenon commonly referred to as asymmetric warming^1^,^2^,^3^,^4^,^5^. These observations are further supported by climate change scenarios predicting faster increases in T_min_ than T_max_ particularly in mid to high northern latitudes and in arid regions^6^,^7^. At the same time, a growing body of evidence from long-term observations^8^,^9^,^10^,^11^,^12^,^13^, manipulation experiments^14^,^15^,^16^,^17^,^18^ and model simulations^19^,^20^,^21^ has demonstrated differential impacts of increases in minimum and maximum daily temperatures on plant productivity and terrestrial ecosystems carbon budgets. However, most experiments have been conducted under diurnal constant (symmetric) warming simulations^22^,^23^, and many models only use daily, monthly, or even annual mean temperatures for the temperature parameterizations when simulating and predicting the responses and feedbacks of terrestrial ecosystems to global warming^24^,^25^. In contrast, few studies have to date been conducted where the differential warming has been explicitly incorporated to examine the impact of the observed asymmetries on terrestrial ecosystem behaviors^20^,^26^,^27^.

The effects of warming on plants and entire ecosystems also depend on interactions with other environmental factors such as precipitation, atmospheric CO_2_ concentration, nitrogen depositions and general nutrient availability^28^,^29^,^30^. In addition, ecosystems located in different climatic zones are likely to respond differently to changes in these factors^12^,^13^,^31^,^32^. It is therefore important to understand the combined effects of asymmetric warming and changes in other environmental variables impact on fundamental metabolic ecosystem processes like photosynthesis and respiration.

Manipulative experiments are key tools to understand the mechanisms of ecosystem responses to climate change^22^. Nonetheless, establishing the impact of asymmetric warming on terrestrial carbon cycling in the field is a key challenge^32^, and it is very difficult to simultaneously simulate the interactive effects of precipitation, elevated CO_2_ and temperature^33^. Ecosystem modeling is therefore highly instrumental to stimulate hypotheses formulation and to extrapolate results from very limited, selected ecosystem settings across ecosystems, wider geographic areas and into the future^28^.

In this study, we use a well-established process-based ecosystem model, Biome-BGC (BioGeochemical Cycles)^34^, to compare the differential effects of symmetric and asymmetric warming on net primary productivity (NPP) and resulting carbon balances of six contrasting ecosystems in northern China. Our main objectives are to determine how plant productivity and ecosystem carbon sequestration are affected by temperature change asymmetries under various environmental change scenarios, and how these responses relate to variations in precipitation and atmospheric CO_2_ concentrations.

## Methods

Ecosystem processes are modelled using the Biome-BGC, which can simulate biogeochemical and hydrological processes of multiple biomes, using daily meteorological data including maximum, minimum and average temperature, precipitation, vapor pressure deficit, daylight average shortwave radiant flux density, and length of the day between sunrise and sunset^34^. Several further variables like the average day-time temperature (T_day_) and average night-time temperature (T_night_) are calculated from recorded maximum and minimum temperatures and meteorological principles^35^, allowing for sunlight-dependent processes like photosynthesis to be driven by T_day_, while processes such as decomposition are driven by 24 h averages. At the same time, maintenance respiration (R_m_) of all living tissues is driven by changing temperature conditions throughout the day. R_m_ is calculated separately for sun and shade leaves and partitioned into night- and daytime respiration, with daytime respiration also needed to calculate net assimilation. R_m_ of sapwood is calculated separately for night and day respiration based on T_night_ and T_day_, respectively. R_m_ of the root system finally is calculated based on the soil temperature, which is assumed to be the 11-day running weighted average of T_day_. Overall, the simulated photosynthesis and respiration processes are sensitive to asymmetric temperature patterns and form the basis for the subsequent model outputs including Net primary productivity (NPP), heterotrophic respiration (R_h_) and net ecosystem production (NEP = NPP − R_h_). We selected a total of three forest and three grassland ecosystems varying in their temperature and precipitation regimes on the north sections of the North-South Transect of Eastern China and the east sections of the China Grassland Transect, respectively^36^ (SI: Figure S1). The Biome-BGC model was adjusted for the six selected sites with a set of site-specific parameters (Table 1). Plant eco-physiological parameters were used according to White *et al.* (2000)^37^, except where detailed site-specific data were available (SI: Table S1). Since the model does not currently simulate mixed forest stands, we divided the temperate mixed forests (TMF) site into evergreen needle-leaf forest (ENF) and deciduous broadleaf forest and simulated them separately^38^. The results were then added given different weights according to the basal area fraction covered by the respective plant functional types^39^ (0.35 for the ENF and 0.65 for the deciduous broadleaf forest, respectively).

Our initial analytical focus was on the differences in ecosystem carbon budgets when comparing symmetric versus asymmetric climate change. For this, we used four different scenarios^20^: ambient scenario corresponding to the historical recorded temperature data during the period of 1961–1990 (T_amb_), symmetric warming (T_sym_), double asymmetric warming (T_asy2_) and triple asymmetric warming (T_asy3_). The three scenarios for temperature increases were based on a combination of recorded recent temperature increases (SI: Figure S2) and the predicted future magnitude of temperature increases simulated by a regional climate model (RegCM3) under the A2 IPCC CO_2_ emission scenarios (SRES A2)^40^ (SI: Figure S3). In the second step, the interactive effects of changes in temperature, precipitation, and atmospheric CO_2_ concentrations were investigated. The precipitation treatment had two levels: an ambient level corresponding to the historical mean precipitation amounts recorded during the period of 1961–1990 (P_amb_), and precipitation change based on the 2071–2100 predictions from the RegCM3 (P_cha_)^40^. The model MT-CLIM (Version 4.3) was used to compute meteorological variables not included in the standard weather station records and required by the Biome-BGC model^41^. The CO_2_ treatment also had two levels: an ambient level corresponding to the historical concentrations recorded during the period of 1961–1990 (C_amb_) based on the Mauna Loa measurements (http://co2now.org/), and a scenario taking into account the gradual predicted increase in atmospheric CO_2_ concentrations from 626 ppm_v_ in 2071 to 836 ppm_v_ in 2100 (C_inc_) as predicted by the SRES A2 emission scenario data^42^.

Analysis of covariance was used to assess the effects of the different temperature treatments on NPP, R_h_ and NEP under the four scenarios, respectively. To avoid over-interpretation of modeled values, rigorous significance tests for the interactive effects of the three factors temperature, precipitation and atmospheric CO_2_ concentration were not attempted. Instead, response patterns of each ecosystem were identified using the method outlined by Luo et al. (2008)^28^.

## Results

### Net primary productivity (NPP)

**Figure 1:**
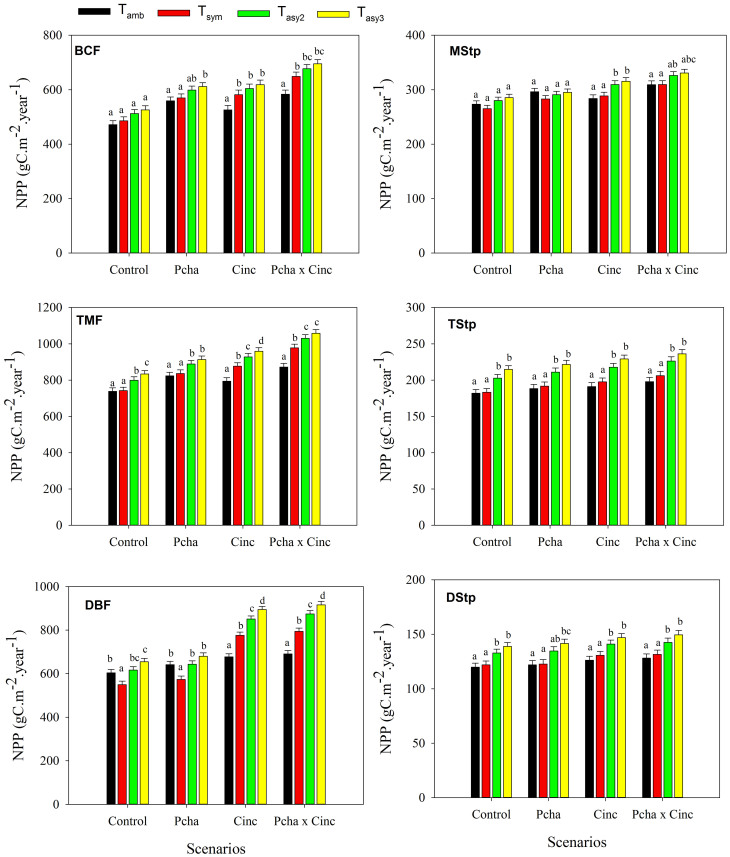
Net primary productivity (NPP) response to the various temperature treatments under four environmental change scenarios, including the control, changes in precipitation amount (P_cha_), gradual increases in concentrations of atmospheric CO_2_ (C_inc_) and their combinations (P_cha_ × C_inc_). Data are means ± standard error, differences letters bars indicate significant (p < 0.05) differences between means. (BCF: Boreal coniferous forest; TMF: Temperate mixed forest; DBF: Warm-temperate deciduous broadleaf forest; MStp: Meadow steppe; TStp: Typical steppe; DStp: Desert steppe)

At most of our study sites, asymmetric warming is predicted to have a significant impact on NPP under the various environmental change scenarios (Figure 1). Under the control scenario, a significantly lower NPP is predicted for T_sym_ than for both T_asy2_ and T_asy3_ scenarios for all ecosystems except for BCF and MStp. Furthermore, significant differences in NPP are computed between T_asy2_ and T_asy3_ scenarios for the two forest ecosystems TMF and DBF.

In scenarios taking into account predicted changes in precipitation (P_cha_), NPP is significantly higher in both T_asy2_ and T_asy3_ in comparison to the T_sym_ scenario for TMF, DBF and TStp, while no significant differences are predicted between T_asy2_ and T_asy3_. For BCF and DStp, NPP predictions are significantly higher for T_asy3_ in comparison to the T_sym_ scenario. By contrast, the different warming treatments has no significant effect on NPP for the MStp.

When increases in CO_2_ concentrations are taken into account (C_inc_ scenarios), NPP shows significant differences between all three warming scenarios for TMF and DBF in the rank order T_asy3_ > T_asy2_ > T_sym_. NPP in T_asy3_ is also significantly higher than in T_sym_ for all three steppe ecosystems. In contrast, no significant changes in NPP for any of the three warming treatments are predicted for BCF.

Under the P_cha_ × C_inc_ scenarios, NPP shows significant differences between all three warming treatments for DBF in the order T_asy3_ > T_asy2_ > T_sym_. NPP is also significantly higher under the T_asy3_ scenario in comparison to T_sym_ for TMF, TStp and DStp. No significant differences between scenarios are recorded for BCF and MStp.

Interactive effects of warming with C_inc_ on NPP are positive at all sites, while the magnitude of these effects varies (Table 2). However, the interactive effects of warming and P_cha_ are negative for DBF, MStp and DStp. The three-way interactions of warming with P_cha_ × C_inc_ are positive for BCF, TMF and DBF. The effects of the remaining two-way and three-way interactions are small in magnitude and not consistent among the three treatments at each site.

### Heterotrophic respiration (R_h_) and Net ecosystem productivity (NEP)

**Figure 2:**
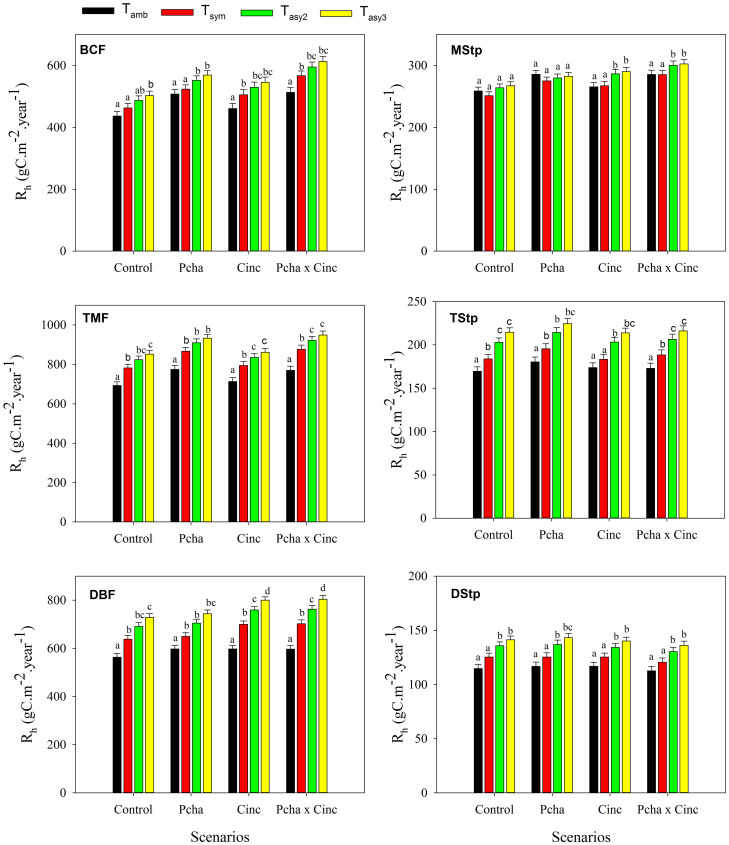
Heterotrophic respiration (R_h_) response to the various temperature treatments under four environmental change scenarios, including the control, changes in precipitation amount (P_cha_), gradual increases in concentrations of atmospheric CO_2_ (C_inc_) and their combinations (P_cha_ × C_inc_). Data are means ± standard error, differences letters bars indicate significant (p < 0.05) differences between means. (BCF: Boreal coniferous forest; TMF: Temperate mixed forest; DBF: Warm-temperate deciduous broadleaf forest; MStp: Meadow steppe; TStp: Typical steppe; DStp: Desert steppe)

Differences between simulated NPP and R_h_ are small when seen in relation to their overall magnitude, and the overall response pattern of modeled R_h_ in the different treatments (Figure 2) is similar to that for NPP. The three-factor combinations of T, C_inc_ and P_cha_ consistently stimulates R_h_, whereas joining temperature regimes individually with either C_inc_ or P_cha_ does not cause consistent response patterns amongst the sites (Table 2).

**Figure 3:**
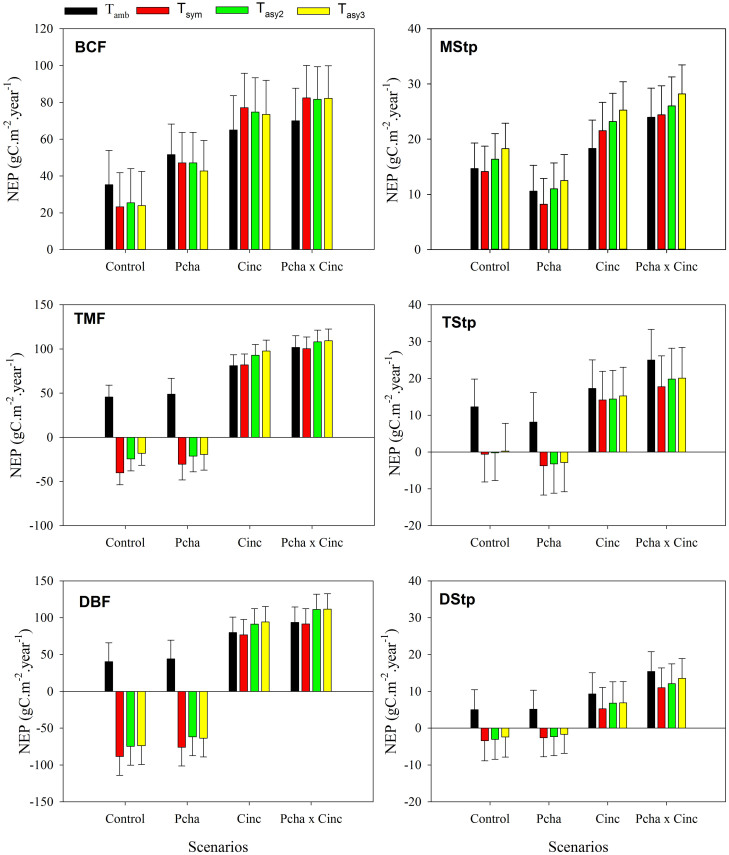
Net ecosystem production (NEP) response to the various temperature treatments under four environmental change scenarios, including the control, changes in precipitation amount (P_cha_), gradual increases in concentrations of atmospheric CO_2_ (C_inc_) and their combinations (P_cha_ × C_inc_). Data are means ± standard error. (BCF: Boreal coniferous forest; TMF: Temperate mixed forest; DBF: Warm-temperate deciduous broadleaf forest; MStp: Meadow steppe; TStp: Typical steppe; DStp: Desert steppe).

The overall response patterns of NEP to the three warming treatments differs strongly to that modelled for NPP and R_h_, with no significant differences resulting for the different temperature treatments under any of the various environmental change scenarios (control, P_cha_, C_inc_, or P_cha_ × C_inc_) (Figure 3).

Similar to the patterns of NPP, the interactive effects of temperature increases with C_inc_ are generally positive for NEP (Table 2). The interactive effects of warming and P_cha_ are positive for BCF, but negative for MStp. The three-way interactions of warming with P_cha_ × C_inc_ are chiefly negative for TMF and MStp. The other two-way and three-way interactions effects on NEP are small in magnitude and highly variable amongst warming treatments for each ecosystem.

## Discussion

In agreement with reports based on historical data analyses^12^ and local experimental observations from the TStp^16^, our model suggests that NPP is significant larger when asymmetries are taken into consideration under various environmental change scenarios at the majority of our study sites. In the BIOME-BGC, day- and night-time warming could have different impacts on the NPP induced by the bias of climate forcing both directly via alterations of leaf processes and indirectly via changes in soil water availability and soil nutrient mineralization rates^34^. This pattern is underpinned by previous modeling simulations^19^,^20^,^21^. All these studies report that asymmetries in climate change patterns have a significant impact on ecosystem productivity, highlighting the great importance to include temperature change asymmetries in future experimental and model studies to realistically project responses and feedbacks of an ecosystem's carbon cycle to climate change^32^,^33^. With photosynthesis occurring during daylight hours and plant and microbial respiration occurring continuously, it could be expected that the latter is much more strongly affected by the strength of asymmetries^8^,^12^,^13^. Nonetheless, our model outputs indicate that NPP and R_h_ show fairly similar response patterns to temperature increases under the various environmental change scenarios at most of the study sites. As a consequence, NEP remains widely unaffected by the degree of asymmetric temperature change in the investigated ecosystems. This result indicates that increases in NPP cannot simply be equated to more carbon sequestration, as other ecosystem processes appear to counter-balance any NEP changes. More importantly, it also strongly suggests that processes of photosynthesis, respiration and carbon sequestration are considered as tightly linked, with photosynthesis and respiration appearing as entities closely coupled through carbon and nutrient supply and demand feedbacks^16^. Daytime warming alters net photosynthesis, which supplies the ecosystem with substrates for respiration at night. Night warming, however, does not only affect night-time ecosystem respiration, but may also stimulate plant compensatory photosynthesis during the following day by the depletion of leaf carbohydrates at night^14^,^16^,^43^. However, like most current biogeochemical models^13^,^32^, BIOME-BGC cannot capture this ‘photosynthesis over-compensation' phenomenon under asymmetric warming due to the missing implementation of the underlying ecophysiological response of plant photosynthesis to nighttime warming through altered draw-down of leaf carbohydrates at night. In addition to the different impacts on plant photosynthesis and ecosystem respiration, day- and night-time warming could have additional impacts on the plant community structure and composition^44^,^45^,^46^, that further impact ecosystem productivity and carbon sequestration^47^,^48^. We therefore suggest that more attention should be paid to the structural and functional responses of carbon-related processes to changes in maximum and minimum day and night temperatures in the current generation of ecosystem models.

Our results indicate simple additive effects of the interactive effects of temperature, CO_2_ concentrations and precipitation are rare, which is consistent with reports based on experiments manipulating temperature and atmospheric CO_2_ concentrations^30^. Overall, single-factor response models may be misleading, creating unreliable predictions of ecosystem responses to multifactorial global change patterns, a trend already observed in temperature-focused experimental studies^16^,^27^. Our study further supports the need for more multifactorial experiments including not only the asymmetric shifts in temperature, but also the influence of precipitation regimes, nutrient availability and atmospheric CO_2_ concentrations to improve predictions of ecosystems responses to global change^29^,^49^ and allow an improved model parameterization and validity.

Models based on the interactions of all three factors considered in our study reveal substantial differences in the magnitude of effects between sites, which somewhat contradicts reports from earlier investigations^28^. This outcome highlights the importance of the local environment and ecosystem structure for the assessment of ecosystem carbon budgets and their response to asymmetric warming^11^,^13^,^32^. While the present analysis was restricted to a limited number of sites focused only on boreal and temperate ecosystems, we acknowledge that particularly the response of tropical and subtropical ecosystems to asymmetric warming is not well researched at present and merits further investigation.

## Author Contributions

S.H. and S.W. planned and conducted the modelling, while S.H., S.W., F.J. and A.J. jointly wrote the manuscript.

## Supplementary Material

Supplementary Information Supplemental Information (SI): Asymmetric warming significantly affects net primary production, but not ecosystem carbon balances of forest and grassland ecosystems in northern China

## Figures and Tables

**Table 1:** Study site characteristics. Meteorological station location coordinates, elevation, and annual average temperature and annual precipitation statistics (means ± SD)

Vegetation type	Site	Location	Annual average temperature (°C)	Annual precipitation (mm)	Soil type (US soil classification-based)	Effective soil depth (m)	Soil texture (%)		
							Sand	Silt	Clay
Boreal coniferous forest (BCF)	Greater Khingan Mountains	50°50′ 121°30′ 826 m a.s.l.	−4.1 ± 0.9	442.8 ± 83.9	Brown coniferous forest soil	0.6	47	35	18
Temperate mixed forest (TMF)	Changbai Mountains	42°24′ 128°05′ 738 m a.s.l.	3.1 ± 0.8	722.4 ± 108.3	Dark brown forest soil	0.6	40	35	25
Warm-temperate deciduous broadleaf forest (DBF)	Dongling Mountains	40°24′ 115°30′ 1250 m a.s.l.	5.3 ± 0.7	575.2 ± 96.5	Mountain brown soil	1.0	20	50	30
Meadow steppe (MStp)	Changling county	44°42′N, 123°45′E, 145 m a.s.l.	6.0 ± 0.8	430.5 ± 107.3	Meadow chernozem	0.5	20	45	35
Typical steppe (TStp)	Duolun county	42°02′N, 116°17′E, 1324 m a.s.l.	2.8 ± 0.9	376.0 ± 70.5	Calcis-orthic Aridisol	0.6	53	27	20
Desert steppe (DStp)	Siziwang Banner	41°46′N, 111°53′E, 1456 m a.s.l.	3.6 ± 0.9	311.6 ± 72.7	Kastanozem	0.4	65	20	15

**Table 2:** Relative strength of two- or three-way interactive effects on net primary production (NPP), heterotrophic respiration (R_h_) and net ecosystem production (NEP)

Vegetation type	Scenarios*	NPP			R_h_			NEP		
		T_sym_**	T_asy2_	T_asy3_	T_sym_	T_asy2_	T_asy3_	T_sym_	T_asy2_	T_asy3_
Boreal coniferous forest (BCF)	P_cha_	−7.5	−2.1	−3.7	−22.4	−10.9	−7.2	53.0	41.2	18.1
	C_inc_	144.8	99.4	77.9	71.4	48.1	41.4	115.7	122.0	96.1
	P_cha_ × C_inc_	27.2	29.5	35.1	49.3	40.7	37.1	−27.5	−18.3	6.6
Temperate mixed forest (TMF)	P_cha_	11.7	6.1	−6.0	3.7	4.3	−0.7	14.5	−0.3	−13.7
	C_inc_	264.1	134.1	91.5	−14.5	−10.5	−12.0	142.8	164.1	161.6
	P_cha_ × C_inc_	29.2	30.0	34.2	35.9	32.9	36.0	−21.3	−14.7	−12.8
Warm-temperate deciduous broadleaf forest (DBF)	P_cha_	−30.2	−41.8	−27.5	−41.8	−24.4	−18.9	13.5	15.6	11.3
	C_inc_	239.7	353.8	265.6	49.8	33.6	37.4	149.4	162.8	167.3
	P_cha_ × C_inc_	32.5	49.3	38.4	54.5	36.3	30.5	−14.2	−6.3	−5.6
Meadow steppe (MStp)	P_cha_	−32.4	−82.2	−76.0	−18.7	−68.1	−65.6	−77.2	−44.0	−43.2
	C_inc_	140.4	151.4	174.1	129.1	156.1	213.3	178.9	141.0	91.7
	P_cha_ × C_inc_	3.6	23.9	20.2	10.7	36.5	29.0	−34.9	−48.8	−27.3
Typical steppe (TStp)	P_cha_	18.6	13.4	1.5	7.3	3.0	−2.8	−15.0	−13.2	−15.4
	C_inc_	94.3	33.4	24.5	−51.1	−25.3	−19.7	30.8	31.5	47.1
	P_cha_ × C_inc_	−2.4	−0.3	−0.4	50.9	21.1	19.4	−1.5	−6.6	−30.3
Desert steppe (DStp)	P_cha_	−31.0	−2.2	6.6	−31.1	−6.5	0.7	−11.8	4.6	15.8
	C_inc_	55.6	24.4	13.8	−32.9	−18.0	−22.9	29.5	26.3	17.3
	P_cha_ × C_inc_	8.6	−3.4	0.3	28.7	13.5	0.9	−2.6	1.3	−1.8

*P_cha_: changes in precipitation amount C_inc_: gradual increases in concentrations of atmospheric CO_2_ (C) and their combinations (P_cha_ × C_inc_).

**Tsym: symmetric warming; Tasy2: double asymmetric warming; Tasy3: triple asymmetric warming.
