# Supplementary material for: Asymmetric warming significantly affects net primary production, but not ecosystem carbon balances of forest and grassland ecosystems in northern China
Source: Sci Rep. 2015 Mar 13;5:9115. doi: 10.1038/srep09115 (PMC4357852; doi:10.1038/srep09115)
Supplement: Supplementary Information — Supplemental Information (SI): Asymmetric warming significantly affects net primary production, but not ecosystem carbon balances of forest and grassland ecosystems in northern China [file srep09115-s1.doc]

Supplemental Information (SI): Asymmetric warming significantly affects net primary production, but not ecosystem carbon balances of forest and grassland ecosystems in northern China

Hongxin ^S^u1, Jinchao Fe^n^g2, Jan C. Axmach^e^r3, Weiguo Sa^ng1^

^1^State Key Laboratory of Vegetation and Environmental Change, Institute of Botany, Chinese Academy of Sciences, Beijing 100093, P.R. China; ^2^ College of Life and Environmental Science, Minzu University of China, 100081 Beijing, P.R. China; ^3^UCL Department of Geography, University College London, Pearson Building, Gower Street, London WC1E 6BT, UK

Correspondence and requests for materials should be addressed to Weiguo Sang （swg@ibcas.ac.cn）

**Additional Details on the Biome-BGC Model**

Biome-BGC calculates primary physiological processes associated with carbon exchanges such as photosynthesis, maintenance respiration (R_m_), growth respiration (R_g_), heterotrophic respiration (R_h_), and the allocation of photosynthetic assimilates, in daily steps. The calculation of photosynthesis is based on the Farquhar biochemical model^1^, taking into account the N content of leaves, the portion of N in Rubisco, and the T_day_ that controls enzyme kinetics^2^. Photosynthesis furthermore depends on the amount of absorbed, photosynthetically active radiation, the availability of CO_2_ and R_m_. For all living tissues, R_m_ is represented by a Q_10_ function of temperature and a linear function of the N content. R_g_ is approximated as representing 30% of the daily allocation of carbon in each compartment as storage for next year’s growth. Rh represents carbon losses due to decomposition of litter and soil organic matter (SOM) via microbial activities. The rate of litter fragmentation is dependent on moisture and temperature conditions of the site. The SOM pools also undergo decomposition constrained by soil water and temperature. The water state variables are dependent on the soil matrix potential, precipitation and the evapotranspiration at the site. Canopy evaporation of intercepted water, transpiration during photosynthesis, and soil evaporation are calculated using a modified Penman-Monteith Equation (PME)^3^. Overall, the simulated photosynthesis and respiration processes are sensitive to asymmetric temperature patterns and form the basis for the subsequent model outputs including NPP, R_h_ and net ecosystem production (NEP as NPP-R_h_).

**Additional Details on Study Area and Sites**

We selected a total of six contrasting ecosystems varying in their temperature and precipitation regimes in northern China as our research subjects. These represent the most important natural vegetation in corresponding climatic zones^4^ (Figure S1). Along the North-South Transect of Eastern China (NSTEC), the three chosen natural forest ecosystems represent a boreal coniferous forests (BCF) dominated by larch (*Larix gmelinii*) on the Greater Khingan Mountain, Inner Mongolia Autonomous Region^5^, a temperate mixed forests (TMF) dominated by *Pinus koraiensis* Sieb. et Zucc., *Tilia amurensis* Rupr., *Quercus mongolica* Fisch. ex Ledeb. and *Fraxinus mandschurica* Rupr. at Changbai Mountain, Jilin Province^6^ and a warm-temperate deciduous broad-leaved forests (DBF) dominated by *Quercus wutaishanica* Mayr in the Dongling Mountains, Beijing^7^.

Along the China Grassland Transect (CGT), the three sites selected along a gradient of decreasing precipitation from east to west^8^ represent a meadow steppe (MStp) dominated by *Leymus chinensis* (Trin.) Tzvel., *Artemisia scoparia* Waldst. et Kit.*, Carex duriuscula* C. A. Mey.*, Kalimeris integrifolia* Turcz. ex DC.*, Phragmites australis* (Cav.) Trin. ex Steud.*,*and *Artemisia anethifolia* Web. ex Stechm at Changling county, Jilin Province^9^, a typical steppe (TStp) dominated by *Stipa krylovii Roshev*, *Artemisia frigida Willd.*, *Potentilla acaulis L.*, *Cleistogenes squarrosa* (Trin.) Keng, *Allium bidentatum*, and *Agropyron cristatum* (L.) Gaertn. in Duolun county, Inner Mongolia Autonomous Region^10^, and a desert steppe (DStp) dominated by *Stipa breviflora* Griseb., *Artemisia frigida* Willd and *Cleistogenes songorica* (Roshev. Ohwi) in the Siziwang Banner, Inner Mongolia Autonomous Region^11^.

According to the historical data from the nearest weather stations for the period 1961–2010 obtained through the China National Climatic Data Center (NCDC), the climate change at all of the study sites was characterized by an asymmetric warming, with the asymmetric trends (ratio) between increases in T_min_ versus T_max_ ranging from 0.89 in the Daxinganling mountain to 2.78 at Changbai mountain (Figure S2). This represents an ideal setting to fully explore the effect of differential warming on the functions of a wide range of ecosystems. Additionally, all six ecosystems have been intensively studied for decades, including experiments of the impact of warming and fertilization at the three grassland sites^10-12^. The information collected from the field sites therefore provide valuable data for model parameterization.

**Additional Details on** **Model Eco-physiological Parameters**

Generic ecophysiological parameters used in Biome-BGC for global biomes are summarized in White et al. (2000)^13^. In this study, plant eco-physiological parameters were updated and adjusted as much as possible (Table S1). The boreal coniferous forest (BCF) site of the Greater Khingan Mountain (GK) is dominated by *Larix gmelinii,* and ecophysiological data for this species was often sparse. Hence, we used some parameter values from *Larix decidua* summarized in Pietsch et al. (2005)^14^ for this plant functional type. The remaining parameters used were default values in the Biome-BGC for the plant functional types as described in White et al. (2000)^13^.

The evergreen needleleaf forest (ENF) of Changbai Mountain (CB) is dominated by *Pinus koraiensis*, and data for species-specific parameters were mainly taken from Cheng et al. (1992)^15^ and Yang & Li (2005)^16^. The deciduous broad-leaf forest (DBF) of CB and Dongling Mountain (DLing) used the same parameter values as previous studies^18^.

The meadow steppe (MStp) of Changling county (CL) is dominated by *Leymus chinensis* and was parameterized based on values published by Dong & Yu (2008)^18^ for this community. Similarly, the typical steppe (TStp) of Duolun county (DLun) dominated by *Stipa krylovii* was parameterized based on values in Dong and Yu (2008)^18^ for this community. Since the dominant plant functional types in the desert steppe (DStp) of Siziwang Banner (SZ) are also C3 grasses, we used the default values in Biome-BGC^13^ for this grass biome, with more specific data for this study region being very sparse.

**Additional Details on Climate Change Scenarios**

The predicted future magnitude of temperature increases were simulated by a regional climate model (RegCM3). Control simulations correspond to the period 1961–1990, while future scenarios were calculated for the period 2071–2100 in correspondence to the A2 IPCC CO_2_ emissions scenarios (SRES A2). Mean changes in monthly temperature in each of the six grid cells including the study areas were illustrated in Figure S3. For the T_sym_ scenario, recorded current daily minimum and maximum temperatures were increased by the same amount based on the predicted increase in the daily mean temperature for 2071-2100 based on the RegCM3 predictions. Based on the historical records (Figure S2), T_asy2_/ T_asy3_ scenarios were generated under the assumption that the minimum temperature would increase two or three times faster than the increase in maximum temperature, respectively, with the overall change in the mean diurnal temperature range approximately equal to the change in the predicted daily mean temperature. Monthly precipitation changes between the future (2071-2100) and the reference (1961-1990) period under the IPCC A2 scenario are illustrated in Figure S4.

**Additional Details on** **Model Simulations**

The Biome-BGC model simulation had two phases. The first phase is the spin-up (self-initialization) simulation, which was based on historical meteorological data from 1961 to 2010, along with site-specific information on plant eco-physiology and environmental factors such as soil depth and geo-location. The atmospheric CO_2_ concentration was set to 294.84 ppmv, approximating the levels at pre-industrial age. The second phase is the ‘normal’ simulation of NPP, R_h_ and NEP responses to the various environmental change scenarios. At each study site, these three variables were estimated for T_amb_, T_sym_, T_asy2_ and T_asy3_ under the four environmental change scenarios: the control scenario keeping precipitation and atmospheric CO_2_ concentrations in correspondence to the historical values recorded during the period of 1961–1990; a scenario for change in precipitation (P_cha_), only; a scenario focusing on the gradual increase in atmospheric CO_2_ (C_inc_); and finally a scenario combining these two factors (P_cha_ × C_inc_).

1. Farquhar, G., von Caemmerer, S. V., Berry, J. A biochemical model of photosynthetic CO_2_ assimilation in leaves of C_3_ species. *Planta* **149**, 78-90 (1980).
2. Woodrow, I. E. & Berry, J. Enzymatic regulation of photosynthetic CO_2_, fixation in C_3_ plants. *Ann. Rev. Plant Physiol. Plant Mol. Biol.* 39, 533-594 (1988).
3. Waring, R. H. & Running, S. W. *Forest ecosystems: analysis at multiple scales (Third Edition)*. (Academic Press, San Diego, 2007).
4. The editorial committee of vegetation map of China, Chinese Academy of Sciences. *Vegetation map of the People’s Republic of China 1:1000000*. (Geological Publishing House, Beijing, 2007).
5. Jiang, Y. & Zhou, G. Carbon balance of *Larix gmelini* forest and impacts of management practices. *Acta Phytoecologica Sin.* **26**, 317-322 (2002).
6. Wang, M., Guan, D., Hao, S. & Wu, J. Comparison of eddy covariance and chamber-based methods for measuring CO_2_ flux in a temperate mixed forest. *Tree physiol.* **30**, 149-163 (2010).
7. Su, H. & Li, G. Simulating the response of the *Quercus mongolica* forest ecosystem carbon budget to asymmetric warming. *Chinese Sci. Bull.* **57**, 1544-1552 (2012).
8. Hu, Z., Yu, G., Fan, J., Zhong, H., Wang, S. & Li, S. Precipitation-use efficiency along a 4500-km grassland transect. *Global Ecol. Biogeogr.* **19**, 842-851 (2010).
9. Wang, Y. & Zhou, G. Modeling responses of the meadow steppe dominated by Leymus chinensis to climate change. *Climatic change* **82**, 437-452 (2007).
10. Wan, S., Xia, J., Liu, W. & Niu, S. Photosynthetic overcompensation under nocturnal warming enhances grassland carbon sequestration. *Ecology* **90**, 2700-2710 (2009).
11. Wang, Z. *et al*. Influence of increasing temperature and nitrogen input on greenhouse gas emissions from a desert steppe soil in Inner Mongolia. *Soil Sci. Plant Nutr.* **57**, 508-518 (2011).
12. Jiang, L., Zhu, T., Ma, L., Shi, L., Hou, W.& Guo, J. Responses of ecosystem’s carbon and water fluxes to global change on the Songnen Steppe. *Sci. Technol. Rev.* **29**, 35-42 (2011).
13. White, M. A., Thornton, P. E., Running, S. W.& Nemani, R. R. Parameterization and sensitivity analysis of the BIOME-BGC terrestrial ecosystem model: net primary production controls. *Earth Interact*. **4**, 1-85 (2000).
14. Pietsch, S. A., Hasenauer, H. & Thornton, P. E. BGC-model parameters for tree species growing in central European forests. *Forest Ecol. Manag.* **211**, 264-295 (2005).
15. Cheng, B., Ding, G., Xu, G. & Zhang, Y. Biological nutrient cycling in Korean Pine-broadleaved forest of the Changbai Mountain. *Res. Forest Ecosyst.* **6**, 185-193 (1992).
16. Yang, L. & Li, W. Fine root distribution and turnover in a broad-leaved and Korean pine climax forest of the Changbai Mountain in China. *J. Beijing Forestry Univ.* **27**, 1-5 (2005).
17. Su, H. & Sang, W. Simulations and analysis of net primary productivity in Quercus liaotungensis forest of Donglingshan Mountain range in response to different climate change scenarios. *Acta Botnica Sin.* **46**, 1281-1291 (2004).
18. Dong, M. & Yu, M. Simulation analysis on net primary productivity of grassland communities along a water gradient and their responses to climate change. *J. Plant Ecol. (Chinese Version)* **32**, 531-543 (2008).

Table S1 Ecophysiological parameters for Biome-BGC

| Parameter | Unit | GK’s DNF | CB’s ENF | CB/DLing’s DBF | CL’s C3 grass | DLun’s C3 grass | SZ’s C3 grass |
| --- | --- | --- | --- | --- | --- | --- | --- |
| Transfer growth period as fraction of growing | *(prop.) | 0.2 | 0.3 | 0.2 | 1 | 1 | 1 |
| Litterfall as fraction of growing season | *(prop.) | 0.2 | 0.3 | 0.2 | 1 | 1 | 1 |
| Annual leaf and fine root turnover fraction | (1/yr) | 1 | 0.26 | 1 | 1 | 1 | 1 |
| Annual live wood turnover fraction | (1/yr) | 0.7 | 0.7 | 0.7 | 0 | 0 | 0 |
| Annual whole-plant mortality fraction | (1/yr) | 0.003 | 0.0025 | 0.005 | 0.1 | 0.1 | 0.1 |
| Annual fire mortality fraction | (1/yr) | 0.000 | 0.000 | 0.000 | 0.000 | 0.000 | 0.000 |
| (ALLOCATION) new fine root C : new leaf C | (ratio) | 1 | 1.4 | 0.8 | 1.5 | 1.5 | 1.5 |
| (ALLOCATION) new stem C : new leaf C | (ratio) | 2.2 | 2.2 | 2.0 | 0 | 0 | 0 |
| (ALLOCATION) new live wood C : new total wood C | (ratio) | 0.071 | 0.071 | 0.15 | 0 | 0 | 0 |
| (ALLOCATION) new croot C : new stem C | (ratio) | 0.23 | 0.29 | 0.23 | 0 | 0 | 0 |
| (ALLOCATION) current growth proportion | (prop.) | 0.5 | 0.5 | 0.5 | 0.5 | 0.5 | 0.5 |
| C:N of leaves | (kgC/kgN) | 27 | 31 | 21 | 13 | 14 | 25 |
| C:N of leaf litter, after retranslocation | (kgC/kgN) | 120 | 90 | 50 | 45 | 45 | 45 |
| C:N of fine roots | (kgC/kgN) | 58 | 38 | 65 | 50 | 50 | 50 |
| C:N of live wood | (kgC/kgN) | 58 | 105 | 78 | 0 | 0 | 0 |
| C:N of dead wood | (kgC/kgN) | 730 | 595 | 410 | 0 | 0 | 0 |
| Leaf litter labile proportion | (DIM) | 0.31 | 0.26 | 0.30 | 0.55 | 0.61 | 0.68 |
| Leaf litter cellulose proportion | (DIM) | 0.45 | 0.49 | 0.44 | 0.38 | 0.33 | 0.23 |
| Leaf litter lignin proportion | (DIM) | 0.24 | 0.25 | 0.26 | 0.07 | 0.06 | 0.09 |
| Fine root labile proportion | (DIM) | 0.34 | 0.25 | 0.29 | 0.34 | 0.34 | 0.34 |
| Fine root cellulose proportion | (DIM) | 0.44 | 0.50 | 0.18 | 0.44 | 0.44 | 0.44 |
| Fine root lignin proportion | (DIM) | 0.22 | 0.25 | 0.53 | 0.22 | 0.22 | 0.22 |
| Dead wood cellulose proportion | (DIM) | 0.71 | 0.71 | 0.66 | 0 | 0 | 0 |
| Dead wood lignin proportion | (DIM) | 0.29 | 0.29 | 0.34 | 0 | 0 | 0 |
| Canopy water interception coefficient | (1/LAI/d) | 0.025 | 0.025 | 0.021 | 0.0225 | 0.0225 | 0.0225 |
| Canopy light extinction coefficient | (DIM) | 0.51 | 0.51 | 0.70 | 0.48 | 0.48 | 0.48 |
| All-sided to projected leaf area ratio | (DIM) | 2.6 | 2.6 | 2 | 2 | 2 | 2 |
| Canopy average specific leaf area (projected area basis) | (m2/kgC) | 22 | 20.2 | 30 | 21 | 15 | 35 |
| Ratio of shaded SLA:sunlit SLA | (DIM) | 2 | 2 | 2 | 2 | 2 | 2 |
| Fraction of leaf N in Rubisco | (DIM) | 0.08 | 0.08 | 0.075 | 0.21 | 0.21 | 0.18 |
| Maximum stomatal conductance (projected area basis) | (m/s) | 0.006 | 0.006 | 0.0065 | 0.006 | 0.006 | 0.006 |
| Cuticular conductance (projected area basis) | (m/s) | 0.00006 | 0.00006 | 0.00001 | 0.00006 | 0.00006 | 0.00006 |
| Boundary layer conductance (projected area basis | (m/s) | 0.09 | 0.09 | 0.01 | 0.04 | 0.04 | 0.04 |
| Leaf water potential: start of conductance reduction | (MPa) | -0.70 | -0.65 | -0.60 | -0.73 | -0.73 | -0.73 |
| Leaf water potential: complete conductance reduction | (MPa) | -2.6 | -2.5 | -2.3 | -2.7 | -2.7 | -2.7 |
| Vapor pressure deficit: start of conductance reduction | (Pa) | 800 | 610 | 930 | 1200 | 600 | 1000 |
| Vapor pressure deficit: complete conductance reduction | (Pa) | 3200 | 3100 | 4100 | 7200 | 4800 | 5000 |

*DNF: deciduous needle leaf forest; ENF: evergreen needle lea forest; DBF: deciduous broad leaf forest.

**GK: Greater Khingan Mountain; CB: Changbai Mountain; DLing: Dongling Mountain; CL: Changling county; DLun: Duolun county; SZ: Siziwang Banner.

Figure S1. The locations of six study sites in northern China on the background of a vegetation map of the China (GK: Greater Khingan Mountain; CB: Changbai Mountain; DLing: Dongling Mountain; CL: Changling county; DLun: Duolun county; SZ: Siziwang Banner). The map was created using ESRI ArcGIS 9.3. The distribution of grassland and forest types is derived from the Vegetation Map of the People’s Republic of China at a scale of 1:1,000,000 , which provided by The editorial committee of vegetation map of China, Chinese Academy of Sciences (2007)^4^.

Figure S2. Time series of annual average maximum temperature (T_max_), minimum temperature (T_min_) and mean temperature (T_mean_) from the nearest weather station at the six sites in northeast China (GK: Greater Khingan Mountain; CB: Changbai Mountain; DLing: Dongling Mountain; CL: Changling county; DLun: Duolun county; SZ: Siziwang Banner). (solid lines represent regression lines)

Figure S3. Changes of temperatures by monthly between the future (2071-2100) and the reference (1961-1990) under the IPCC A2 scenario using RegCM3 for six sites in the northern China (GK: Greater Khingan Mountain; CB: Changbai Mountain; DLing: Dongling Mountain; CL: Changling county; DLun: Duolun county; SZ: Siziwang Banner).

Figure S4.Changes of precipitation by monthly between the future (2071-2100) and the reference (1961-1990) under the IPCC A2 scenario using RegCM3 for six sites in the northern China (GK: Greater Khingan Mountain; CB: Changbai Mountain; DLing: Dongling Mountain; CL: Changling county; DLun: Duolun county; SZ: Siziwang Banner).

Figure S1


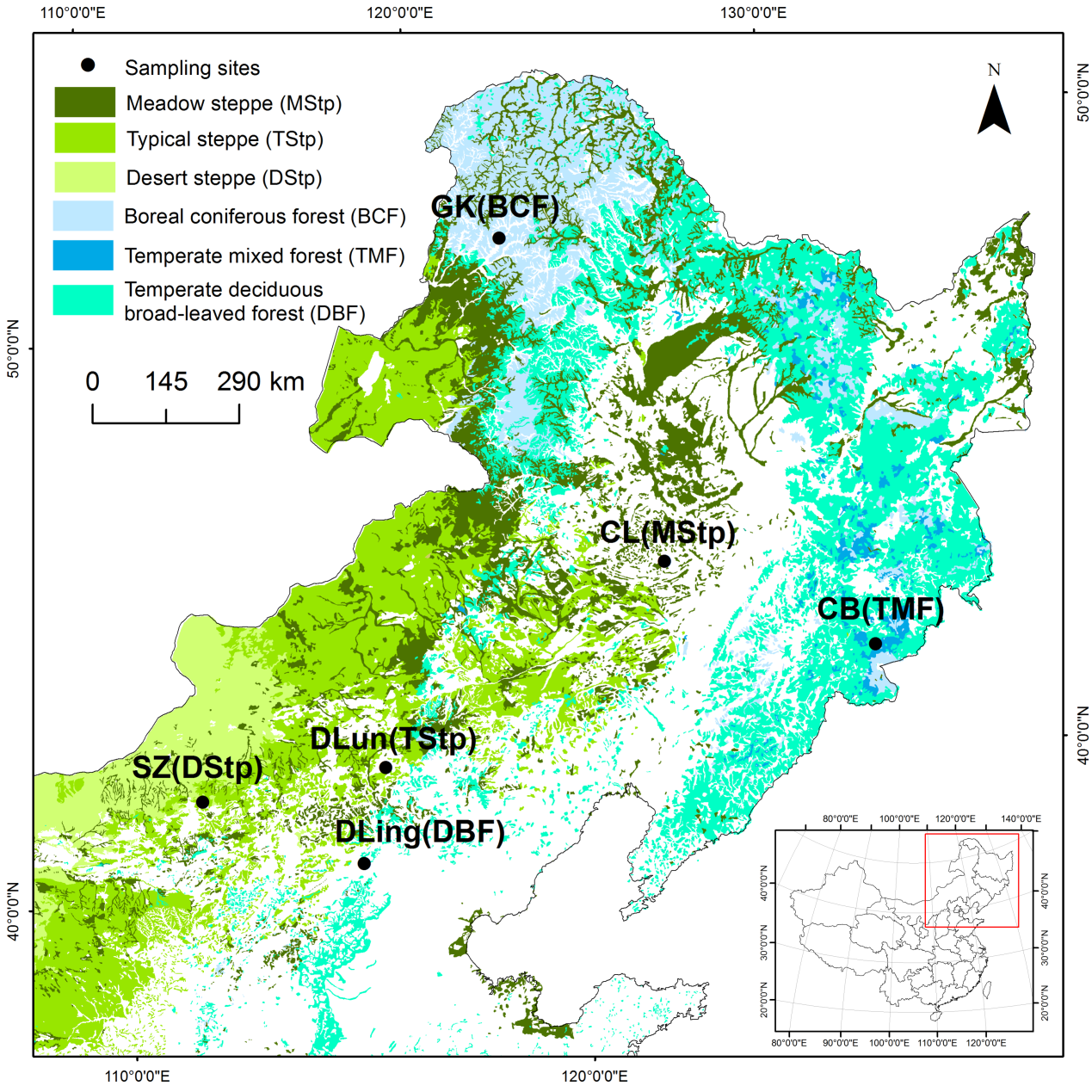


Figure S2

Figure S3

Figure. S4
